# Neuronal Nicotinic Receptors Are Crucial for Tuning of E/I Balance in Prelimbic Cortex and for Decision-Making Processes

**DOI:** 10.3389/fpsyt.2016.00171

**Published:** 2016-10-14

**Authors:** Elsa Cécile Pittaras, Alexis Faure, Xavier Leray, Elina Moraitopoulou, Arnaud Cressant, Arnaud Alexandre Rabat, Claire Meunier, Philippe Fossier, Sylvie Granon

**Affiliations:** ^1^CNRS 9197, Institut de Neuroscience Paris Saclay, Orsay, France; ^2^Institut de Recherche Biomédicale des Armées et Unité Fatigue & Vigilance, Brétigny-sur-orge, France; ^3^Brain@vior, Saint-Prest, France

**Keywords:** brain activation, cfos, prefrontal cortex, gambling behaviors, risk-taking, anxiety, social behavior

## Abstract

**Rationale:**

Decision-making is an essential component of our everyday life commonly disabled in a myriad of psychiatric conditions, such as bipolar and impulsive control disorders, addiction and pathological gambling, or schizophrenia. A large cerebral network encompassing the prefrontal cortex, the amygdala, and the nucleus accumbens is activated for efficient decision-making.

**Methods:**

We developed a mouse gambling task well suited to investigate the influence of uncertainty and risk in decision-making and the role of neurobiological circuits and their monoaminergic inputs. Neuronal nicotinic acetylcholine receptors (nAChRs) of the PFC are important for decision-making processes but their presumed roles in risk-taking and uncertainty management, as well as in cellular balance of excitation and inhibition (E/I) need to be investigated.

**Results:**

Using mice lacking nAChRs – β2^−/−^ mice, we evidence for the first time the crucial role of nAChRs in the fine tuning of prefrontal E/I balance together with the PFC, insular, and hippocampal alterations in gambling behavior likely due to sensitivity to penalties and flexibility alterations. Risky behaviors and perseveration in extinction task were largely increased in β2^−/−^ mice as compared to control mice, suggesting the important role of nAChRs in the ability to make appropriate choices adapted to the outcome.

## Introduction

Decision-making is an essential component of our everyday life. According to Doya (1), decision follows four steps: recognizing the situation of decision, evaluating the possible options (valuation), selecting the appropriate action in inhibiting all other non-optimal ones (action selection), and eventually learning about this action in evaluating the output (learning). These processes are modulated by various factors, such as motivational internal state, risk, and uncertainty. Studying the part of valuation in decision-making might be achieved by modifying the value of each option using devaluation procedures or by changing their relative quantity or quality (2). Ability to inhibit non-optimal action could be revealed using reversal and/or extinction procedures that require adaptation to a novel rule (3). Finally, the influence of uncertainty and risk-taking in decision-making can be challenged with gambling tasks initially developed in humans, and recently adapted for rodents (4–8).

At a neurobiological level, making decision requires cortico-striatal loop activation that might be separated in a limbic (affective/emotion) and a cognitive loop (executive/motor). The limbic loop would encompass the orbitofrontal cortex, the amygdala and the nucleus accumbens (NAcc), and the cognitive loop would be composed of the prelimbic, infralimbic and anterior cingulate cortices, and the dorsal striatum (9). The limbic loop would participate in evaluation of behavioral outcomes in term of cost, risk, and amount (9) while the cognitive loop would rather play a role in selecting and adapting behavioral choice in regard to change. When facing high uncertainty and risk like in gambling tasks or in social situations, there is an involvement of both loops (9, 10). Additional pieces of recent evidence report the implication of the insular cortex in decision-making under risk or uncertainty (11) and in the development of compulsive behaviors (12). Multiple neuromodulators, such as dopamine, noradrenaline, and serotonin, are highly involved in these loops and affect various components of the decision-making process (1, 4, 9). At a cellular level, decision-making processes are suggested to require a precise control of the E/I balance within cortico-striatal circuits (13, 14). In a recent rat study, modulation of GABAergic function within the medial prefrontal cortex (PFC) has been demonstrated to modulate decision-making in a gambling task (15).

In numerous psychiatric pathologies, alteration of processes involved in decision-making leads to maladaptive choices. These disabilities might underpin behavioral defects in many psychiatric disorders, such as bipolar and impulsive control disorders (16, 17), addiction, or pathological gambling (18, 19). Elevation in the E/I balance within cortico-striatal circuits has been associated with many of these pathologies (14, 20). Indeed, alteration of the PFC E/I ratio has been proposed to trigger cognitive and social dysfunctions in pathologies, such as autism and schizophrenia (20–22). Better knowledge about factors which could influence E/I balance within cortico-striatal circuits and its impact on decision-making abilities is therefore crucial.

The major neuronal nicotinic receptors – nicotinic acetylcholine receptors (nAChRs) – are pentameric oligomers composed of subunits, principal combinations of which are α4β2 subunits, for heteromeric ones, and α7 subunits for homomeric ones (23, 24). Endogenous acetylcholine (ACh) modulates numerous neurotransmitters release in these cortico-striatal circuits via its binding onto nAChRs presynaptically located on dopaminergic, noradrenergic, and serotonergic terminals (25). β2^−/−^ mice (null mice for nAChRs containing the beta2 subunit) exhibited marked alteration in exploration and navigation (26, 27), and in organization of social behaviors, reflecting behavioral flexibility troubles (28–30). In the PFC, both functional β2-nAChRs and monoaminergic inputs are necessary for showing organized social behaviors (28, 31). Previous alteration in β2^−/−^ mice have been reported in a social decision-making tasks in which natural rewards like food, novelty seeking, and social contact compete, with a high level of uncertainty associated to a social conspecific having, by nature, unpredictable behavior (29, 32, 33). By contrast, when such competition existed without uncertainty β2^−/−^ mice were not impaired and exhibited normal choices (33). This highlighted the crucial importance of uncertainty in decision-making for β2^−/−^ mice. In addition, as β2-nAChRs are crucial for PFC activity (28, 34), it is relevant to question their putative implication in the PFC E/I balance. To date, we lack information on β2^−/−^ abilities in complex decision-making with high risk and/or under uncertainty aside from social situations.

In this framework, our current aim is to test if β2-nAChRs could be one of the actors influencing the excitation/inhibition balance within the PFC. Besides, we address the selective role of these receptors in behavioral tasks that target different aspects of decision-making processes: a gambling task that involves uncertainty and risk management, and a novel decision-making task that involved the valuation and devaluation of various outcomes – social, food, and novelty – and which allowed us to investigate behavioral extinction. Finally, we measured cFos expression in multiple brain structures following the gambling task completion.

## Materials and Methods

### Animals

In all the behavioral experiments, male C57Bl/6J mice and β2^−/−^ knockout mice, bred in Charles’ River facilities (L’Arbresle Cedex, France) were used. β2^−/−^ knockout mice were generated from a 129/sv Embryonic Stem line as previously described (35) and back crossed onto the C57Bl/6J strain for 20 generations. As they were shown to be at more than 99.99% C57Bl/6J by a genomic analysis using 400 markers, C57Bl/6J mice were used as control of β2^−/−^ knockout mice. Mice were housed in a temperature controlled room (21 ± 2°C) with a 12 h light/dark cycle (light on at 8:00 a.m.). All experiments were performed during the light cycle between 9:00 a.m. and 5:30 p.m. All experimental procedures were carried out in accordance with the EU Directive 2010/63/EU, Decree N 2013-118 of February 1, 2013, and the French National Committee (87/848).

### Experiment I. Electrophysiological Study of the Excitation/Inhibition Balance in the PFC

In order to better apprehend how lack of β2 subunit in β2^−/−^ animal modulate the activity of prefrontal cortex, we investigated the specific roles of α4β2 or α7 nAChRs in the activity of PFC. For that, we determined the balance between E–I balance inputs onto the soma of L5PyNs and checked the effects of α4β2 or α7 antagonists on E–I balance. Experiments were done both on 67 C57Bl/6 mice and on 38 β2^−/−^ mice from post-natal days 20–25. Electrophysiological study of the PFC was done following the methods extensively described elsewhere (36–38). Briefly, electrical stimulations (1–10 μA, 0.2 ms duration) were delivered in layer 2–3 or in layer 6 using 1 MΩ impedance bipolar tungsten electrodes (TST33A10KT; WPI). Evoked synaptic responses recorded in L5PyNs were measured and averaged at several holding potentials. I–V relationship was then determined at each time-point of the response. An average estimate of the input conductance waveform of the cell was calculated. The decomposition of this input conductance in its excitatory and inhibitory components enables to assess the E–I balance.

The α4β2 antagonist Dihydro-β-erythroidine (DhβE) hydrobromide (Sigma), and the α7 antagonist methyllycaconitine (MLA, Tocris) were perfused in the bath solution for at least 15 min before recording.

### Experiment II. Mouse Gambling Task

Our aim in this task is to test the gambling profile of β2^−/−^ knockout mice and how their brain were activating during the gambling task using cellular imaging with c-fos immunohistochemistry.

Twenty-four male C57Bl/6J and 21 β2^−/−^ mice of 3–6 months old were used. Mice were group-housed (three or four mice per cage) and were food deprived (maintenance at 85% of the free feeding weight) with water *ad libitum*.

#### Behavioral Procedures of the Mouse Gambling Task

This decision-making task inspired by the human Iowa Gambling Task (39) was previously adapted to mice (4, 8).

##### Habituation in Operant Chambers

Mice were habituated to be manipulated by experimenters, to eat pellets, and to make an effort to get food pellets in operant chambers for 10 days before starting the mouse gambling task (MGT). The central hole was the only hole available. A nose poke led to distribution of one food pellet in the magazine. After consumption a fixed 5-s delay occurred before which a new trial began. The daily session continued until 65 pellets were obtained or for 30 min, whichever arrived first.

##### Mouse Gambling Task Apparatus and Protocol

The task took place in a maze with four transparent arms (20 cm long × 10 cm wide) containing an opaque start box (20 cm × 20 cm) and a choice area. We used standard food pellets as a reward (dustless Precision Pellets, Grain-based, 20 mg, BioServ^®^, New-Jersey) and food pellets previously steeped in a 180 mM solution of quinine as penalty (7, 8). The quinine pellets were unpalatable but not uneatable. Each mouse performed 10 trials in the morning and 10 trials in the afternoon during 5 days, i.e., 100 trials at the end of the experiment.

**Figure 1:**
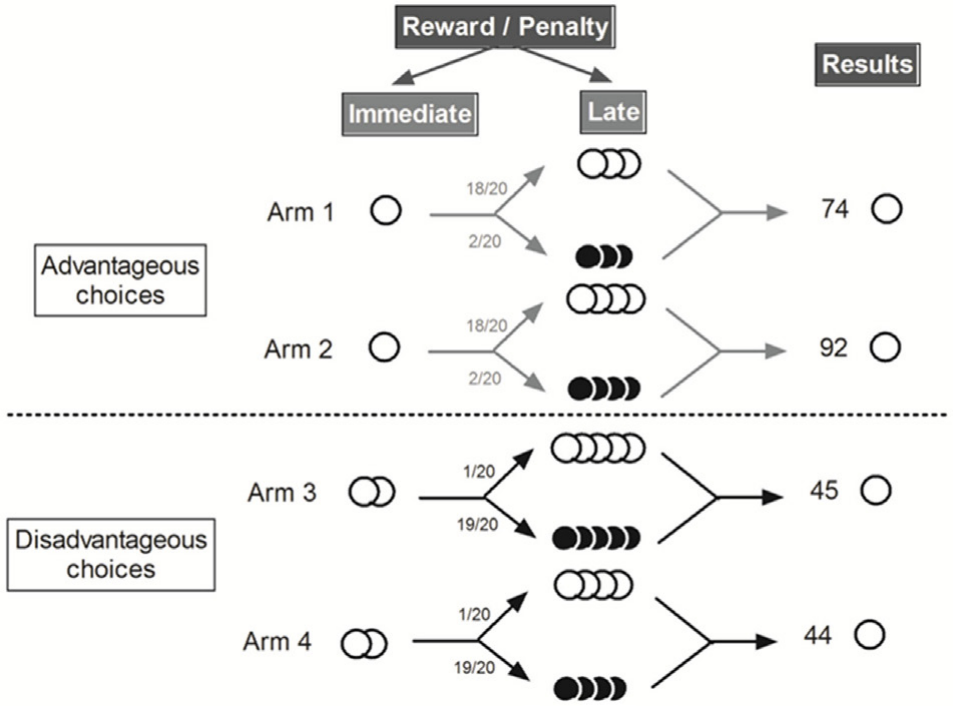
**Schematic representation of the MGT experimental design [Adapted from Ref. (8)]**. White circle represented food pellets and black circle quinine pellets. “Advantageous” choices gave access to one food pellets and then to 3 or 4 food pellets (18/20) or quinine pellets (2/20). “Disadvantageous” choices gave access to 4 or 5 food pellets (1/20) or quinine pellets (19/20). We distinguished “advantageous” choices from “disadvantageous” ones because mice earned more pellets (74 or 92 pellets vs. 45 or 44 pellets) after 20 trials by choosing the “advantageous” ones.

Two of the four arms gave access to “advantageous” outputs: immediate access to a small reward, represented by 1 pellet, followed by additional small rewards (3 or 4 pellets) 18 times out of 20 and two times out of 20 by small penalty (3 or 4 quinine pellets). The two other arms gave access to “disadvantageous” outputs: immediate access to 2 pellets followed most of the time by 4 or 5 quinine pellets (19 trials out of 20) or large reward (4 or 5 pellets) one trial out of 20. Despite the immediate less attractive amount of reward “*advantageous*” choices are, thus, more advantageous in the long term and “*disadvantageous*” choices are less advantageous in the long term (Figure 1). Mice had, thus, to favor small immediate reward (“*advantageous”* choices) to obtain the largest amount of pellets at the end of the day.

Between each trial, the maze was cleaned up with distilled water; and between each mouse, it was cleaned up with a 10% of alcohol solution. During the first session, animals were put into the maze during 5 min with food pellets scattered everywhere (habituation). If mice did not eat any food pellets during the first habituation in the morning, a second 5 min habituation period was conducted during the afternoon. For the following sessions, habituation lasted only 2 min without food pellets on. At the beginning of each trial, the mouse was placed in an opaque tube in the starting box to avoid directing the future choice of the animal. After 5 s, we removed the opaque tube and let the animal freely choosing one arm of the maze.

We measured the time spent by the mouse to choose one arm (i.e., when the animal crossed 1/3 of the arm) and we scored the arm chosen and the pellets consumption (pellets earned).

What we call the rigidity score of an animal is the highest percentage of choice of an arm during this period. The first step is to calculate the percentage of choice in all four arms in regard to the total number of possible choices. In first two gambling sessions, an animal get 40 possible choices. If he choose 21 times the arm 1, the score for this arm will be [(21/40) × 100] = 52.5%, 9 choices for arm 2 [(9/40) × 100] = 22.5%, 4 choices for arm 3 [(4/40) × 100] = 10%, and 6 choices for arm 4 [(6/40) × 100] = 15%. Thus, rigidity score of this mouse in these 2 days of gambling is the maximal percentage of choice, i.e, 52.5%. For example, the rigidity score was 25% if animals chose equally advantageous options and disadvantageous ones. A 50% score reflected that animals chose twice more one arm than the others and a 75% score that animals have chosen one arm 3 times out of 4.

In summary:

– A small reward was available at all time in all arms.
– All mice performed 100 trials.
– The four arms had specific contingencies that cannot be predicted because they are not fixed but probabilistic.

The data are shown as percentage of “advantageous” choices that encompass choices made on the two advantageous arms.

###### Subgroups Formation

To built subgroups of choices, we calculated the mean of the 30 last trials (i.e., when performance was stable and strategies established) and we used the k-mean clustering separation using Statistica software (version12) (40). Each animal belonged to a set that had the closest mean to its own performance value. As such, animals were separated on three groups: those which made a majority of advantageous (safe) choices at the end of the experiment, called “safe”; those which maintained some visit in the disadvantageous arms until the end of the experiment, called “risky”; those which had an intermediate behavior, with a majority of choices in the advantageous arms but some unfrequent visit of risky options, called “average.” For each mouse, we calculated a rigidity score at the beginning (two first days) and at the end (two last days) of the experiment.

#### C-fos Immunohistochemistry

The brains of WT mice (*n* = 24) and β2^−/−^ mice (*n* = 11) that have done the MGT were analyzed for c-fos immunohistochemistry.

##### Brains Removed and Conservation

Animals were anesthetized [for 2 ml: Rompun 2%, 50 μl; Kétamine 500, 600 μl; phosphate buffered solution (PBS) 1×, 1350 μl. 1 ml for 10 g] exactly 90 min after the end of the last MGT trial of the week. This timing allows the synthesis of c-fos (early immediate gene) protein in the nuclei of activated neurons. Then, mice were perfused transcardially with 20 ml (PBS) and then by 50 ml of 4% paraformaldehyde (PFA). Brains were removed, fixed during 24 h with PFA and cryoprotected with croissant sucrose solution during 3 days at 4°C. Then brains were put in −20°C in glycerol.

##### Brains Slices and Immunohistochemistry

Brains were sliced with a vibratome (Leica, VT1000E) on a coronal plane into 40 μm. After between two 4 × 10 min rinses in PBS, endogenous peroxidases were neutralized during 30 min in PBS containing 3% H_2_O_2_. To block the non-specific site, we used PBS solution with 1% bovine serum albumin (BSA), 3% normal goat serum (NGS), and 0.2% Triton ×100 during 2H. c-fos immunolabeling was performed with a purified polyclonal rabbit IgG anti-human c-fos [anti c-fos (Ab-5) (4-17) rabbit pAb, CALBIOCHEM] diluted 1:20.000 in 1% BSA, 3% NGS, and 0.2% Triton x100 during 38H. After 4 × 10 min rinses in PBS, sections were incubated for 2H with secondary biotinylated antibody (Biotin Goat anti-rabbit IgG (H + L), INTERCHIM) diluted 1:2.000000 in 1% BSA, 3% NGS, and 0.2% Triton ×100 during 2H. After 4 × 10 min rinses in PBS, the staining was revealed using H_2_O_2_ and diaminobenzidine (D-5905, SIGMA) for 3 min. After rinsing, sections were flattened on SuperFrost glass slides (Menzel-Gläser, Braunschweig, Germany), dehydrated with xylene, and mounted with Eukitt solution.

##### Images Acquisition and Quantification of c-Fos^+^ Nuclei

Quantification was performed by identifying spot positions. c-Fos^+^ were counted with ICY software (http://icy.bioimageanalysis.org/) after acquired images using a digital camera (Nikon DXM 1200) of an Olympus BX600 microscope coupled to a software (Mercator Pro; Explora Nova, La Rochelle, France). The constant use of a X10 Plan Apo objective allowed to have a good resolution for c-fos immunochemistry. The focus was set on the upper face of each section before digitization. Each region of interest (ROI) was delimited on the screen for each picture based on the mouse atlas (41). ICY software directly counts the number of cells in the ROI. Cell density per square micrometer was thereafter calculated. The ROIs chosen included the prelimbic (PrL), infralimbic (IL), orbitofrontal lateral, median, dorsolateral and ventral cortex (OFC), the NAcc, caudate putamen (CPu), basolateral nucleus of amygdala (BLA), the hippocampus (Hipp), motor cortex (M) and agranular and granular insular cortex, and dorsal and ventral (CIns). Figures 7, 8, and 9 from the atlas were chosen to analyze PrL and OFC. Figures 17, 18, and 19 were chosen to analyze PrL, IL, Cg, M, CIns, NAcc, and CPu and Figures 41, 42, and 43 to analyze BLA, Amy (amygdala), and H (hippocampus).

### Experiment III: Valuation and Inhibition Processes in a Decision-Making Task with Three Concurrent Motivations. (Explicit Choice, Motivational Modulation of Explicit Choice, Change in Rule)

**Figure 2:**
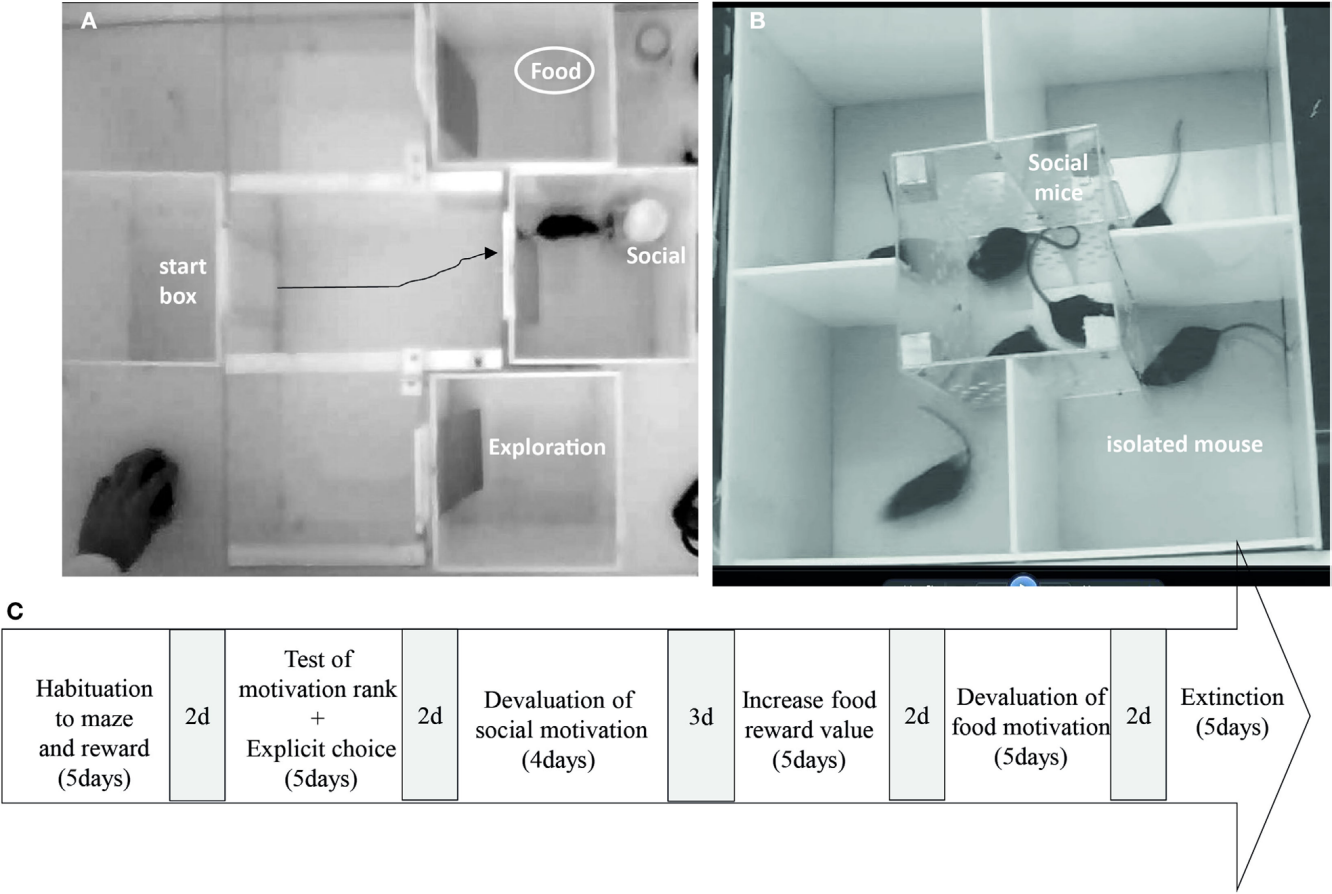
**(A)** Maze choice task apparatus and **(B)** social devaluation cage during the explicit choice task. **(C)** Time schedule of the explicit choice task. 2d or 3d in gray represent days during which animals remained quietly in their home cage.

We first aimed at testing whether β2^−/−^ mice are able to rank efficiently competing rewards and to make choice when no uncertainty/risk is associated. Second, we tested their ability to adapt and modulate their choices as a function of the nature and the value of the reward, or when the rule change in extinction (for time schedule, see Figure 2C).

#### Animals

Eight C57Bl/6J male mice and 8 β2^−/−^ male mice were used for the task. Animals were 8 weeks old at their arrival in the colony room (obtained from Charles River, L’Arbresle Cedex, France). Two weeks arrival, animals underwent 3 weeks of social isolation before the first step (Figure 2A). They underwent a small water restriction in order to increase their motivation for food and water retrieval. Water restriction was established as follow: 24 h total restriction, 3 days with 4 h/day access to water, 12 days with 1 h access to water, 12 days with 30 min access to water, and eventually 25 days with 15 min access to water. During water restriction, animal’s weight progressively decreased to 95% of the free feeding weight and came back to 98–100% at the end of the procedure. An additional group of C57Bl/6J group-housed (four or five mice per cage) male mice (*n* = 18) were used as social reward in the behavioral tasks. These “social” mice were age related with the isolated mice and were given food and water *ad libitum*. All experiments were performed during the light cycle (from 9:00 a.m. to 6:00 p.m). The general health of isolated mice was regularly checked, and body weights were assessed every day throughout the experimental period.

#### Apparatus

The maze (Figure 2A) consisted of four identical opaque Plexiglas boxes with a front sliding door, a flexible plastic door, and a transparent Plexiglas arena (L: 22 cm × l: 61 cm × H: 24 cm). One of the opaque Plexiglas box was used as a start box which opened on the transparent arena, and the three other boxes were goal boxes also connected to the transparent arena with door set equidistant to the start box door (30 cm). Once the mouse was released from the start box, it could roam in the arena and reach one of the goal boxes. To avoid the view of the reward, we inserted a flexible plastic door that animals could easily push to enter the box. Light levels of boxes were set around 25–30 Lux and that of the arena at ~35 lux. Social mice were placed under a large cup (L: 7 cm × l: 7 cm × H: 10 cm) containing holes (0.8 cm diameter), so that animals could smell and touch each other. Food reward was placed in food cup (5.5 cm in diameter, 1 cm high).

#### Behavioral Protocols

##### Explicit Choice

In this part of the protocol, we aimed at assessing how β2^−/−^ mice organized their explicit choices between each reward. For that, we first scored the latency to collect reward as an index of motivation, and then we tested their choices between each reward.

Animals were taken out of the animal facility by group of four animals (2 C57Bl/6J and 2 β2^−/−^) and stocked in the maze room on a nearby table during 15 min before the beginning of the test. Food reward consisted of 15 μl of 0.1% liquid saccharin (0.1 g saccharin sodium salt hydrate from Sigma in 100 ml water) in a cup in the food reward box and social reward consisted of a 20-s contact with a social mouse restrained under the cup in the social reward box (only nose–nose contact was allowed). For each kind of trial, the four animals were put successively in the maze. Social mice were habituated to mild restriction under the cup in a 5-min session in another box before been gently placed in the social reward box. The third reward box simply consisted of an empty box allowing novelty exploration.

###### Habituation to Maze and Reward (5 Days)

Mice were individually placed in the maze for a 10-min habituation session during two consecutive days. All doors of goal boxes were maintained opened but they contained no reward. To avoid potential neophobia mice received 2 ml of 0.1% saccharin in their home cage during these two first days. On day 3, isolated animals were habituated to reward consumption in the maze. For each animal, each reward was permanently assigned to a precise goal box (food, social, and novelty exploration) and position of the reward in the goal boxes were counterbalanced among groups. During this reward habituation days, animals were submitted to six trials, two trials per reward. In each trial, animals were directly placed in a goal box with reward (either access to 15 μl liquid saccharin until full consumption, 20 s access to a social mouse and 20 s in a novel empty box). The fourth day, animal were submitted to a 15-min free choice habituation paradigm, during which *ad libitum* rewards (food: 8 ml 0.1% saccharin, social mouse under a cup, and empty novel box) were available and all goal boxes opened. Social reward was provided by a novel mouse.

###### Reward Ranking (4 Days)

After 2 days off, we begin 4 days of forced choice in order to collect the latency to reach each reward. Each day, mice were submitted to 12 forced choice trials were during which they had to enter one of the three goal box to get the reward (4 trials of food followed by 4 trials of social and 4 trials of exploration). Each trial started by 10 s in the start box before the door was opened and the mouse allowed entering the central arena. If the mouse was not exiting the start box for 30 s, it was gently pushed in the central arena and the sliding door was closed. During these trials, only the door of the target reward was open. Once the mouse entered the goal box, the sliding door was manually closed. If the mouse failed to enter the goal box in 60 s, it was removed from the maze and the trial ended. At the end of the trial, the mouse was put back in its home-cage. Between each trial, the maze was cleaned with tap water in order to homogenize odors. The order of the four trials was randomized during the 4 days. In this part, we online measured the latency of to reach goal boxes.

###### Explicit Choices (1 Day)

In the following day, animals were given a choice between the three rewards at each trial. For each 12 trials, animals had to choose between one of the reward (food, social, and novelty exploration). Once entered in a chosen goal box, the sliding door was manually closed and the animal could consume the reward for 20 s. The maximum choice latency was set at 180 s. The same social mouse was used during four choice trials of the four animals in the group i.e., for a total of 16 trials and ~15–20 min.

##### Motivational Modulation of Explicit Choice

In this part, we aimed at testing adaptation of β2^−/−^ mice choices when social or food reward value was modulated.

###### Devaluation of Social Reward (4 Days)

After 2 days off, we submitted all animals to a devaluation of the social reward. Devaluation of social reward is achieved in inducing social reward “satiety” in mice with 1 h exposure to social reward. More precisely, on social devaluation day (D), all animals were first put by four during 1 h in a devaluation cage placed in the maze room, with three mice in the middle and available nose–nose social contact (Figure 2B). Immediately after, they will be tested in explicit choice protocol (12 trials) with these social mice as social rewards. During control day of social non-devaluation (ND), all animal were put in the maze room in a cage for 1 h, resulting in no social reward “satiety.” Immediately after, they will be tested in explicit choice protocol (12 trials). The social devaluation day (D) preceded the non-social devaluation day (ND) and on the two following days, called postD1 and postD2, animals were submitted to 12 trials of explicit choices.

###### Increase Food Reward Value: Change of Saccharin Quality and Quantity (5 Days)

After 3 days off, animals were submitted to free choices protocol for 1 day, and then on the next day, we exposed to a change of food reward from 1 drop of 0.1% saccharin to 2 drops of 1% saccharin. During this reward habituation day, animals were submitted to six trials (two trials by reward) as exposed above. This novel food reward was maintained for the rest of the experiment.

###### Devaluation of Food Motivation (5 Days)

In order to test the impact of food reward devaluation, we pre-exposed the mice to food reward *ad libitum* (8 ml of 1% liquid saccharin) in the maze room for 1 h before the free choices protocol. In the non-devalued control condition, the same was done but the food cup was empty. Animals were thereafter exposed to 5 days of free choices. For the first day, we followed a classical free choices protocol. On day 2, half of the mice were submitted to devaluation of food motivation (devalued) and the other half to the not-devalued procedure. On day 3, we followed a classical free choices procedure to minimize possible long-lasting impact of *ad libitum* consumption of 1% liquid saccharin. On day 4, we alternated the animals that were devalued or not. On day 5, we followed the classical free choices protocol.

##### Adaptation to a Change of Rule

###### Extinction (5 Days)

After 2 days off, animals were submitted to an extinction protocol. Extinction consisted of presentation of no reward in any goal boxes. Animals were left 20 s in the chosen box.

We measured the number of choices made in each goal boxes, the choice latency to enter goal boxes, and the number of social contacts done.

### Statistical Analysis

#### Experiment I

Differences between means were evaluated for statistical significance using the *t*-test for paired and unpaired conditions samples and the Mann–Whitney U-test when data would not follow a normal law of distribution.

#### Experiment II

When considering all animals (i.e., before subgroup separation), we used ANOVAs usingVAR3 statistical software (42) with an alpha level of 0.05. In order to test global differences from chance level (50%) we use Wilcoxon rank sum test, paired version (*Z* of the Wilcoxon test is displayed in Statistica software). Once subgroups were made and number of animals was below 30, we considered that data would not follow a normal law of distribution. We, thus, used Mann–Whitney or Kruskal–Wallis non-parametric test when appropriate.

#### Experiment III

Non-parametric analyses were performed using R software (version 2.13.2 (2011-09-30) copyright (c) 2011 the R foundation for Statistical computing with Rcmdr-package), as some of the scored behavior would not follow a Gaussian distribution. We used Wilcoxon rank sum test for two samples, the Wilcoxon-signed-rank test for paired data, and Friedman chi-squared test.

## Results

### Experiment I

#### Beta2-nAChRs Are Necessary for the Regulation of the Prefrontal E/I Balance

To determine the role of nAChRs in the PFC cellular activity, we determined the balance between E–I balance inputs onto the soma of layer 5 pyramidal Neurons (L5PyNs) and we checked the effects of α4β2 or α7 antagonists on this E–I balance. This strategy permitted to analyze the role of endogenous release of ACh on the activity of cortical excitatory and inhibitory networks.

**Figure 3:**
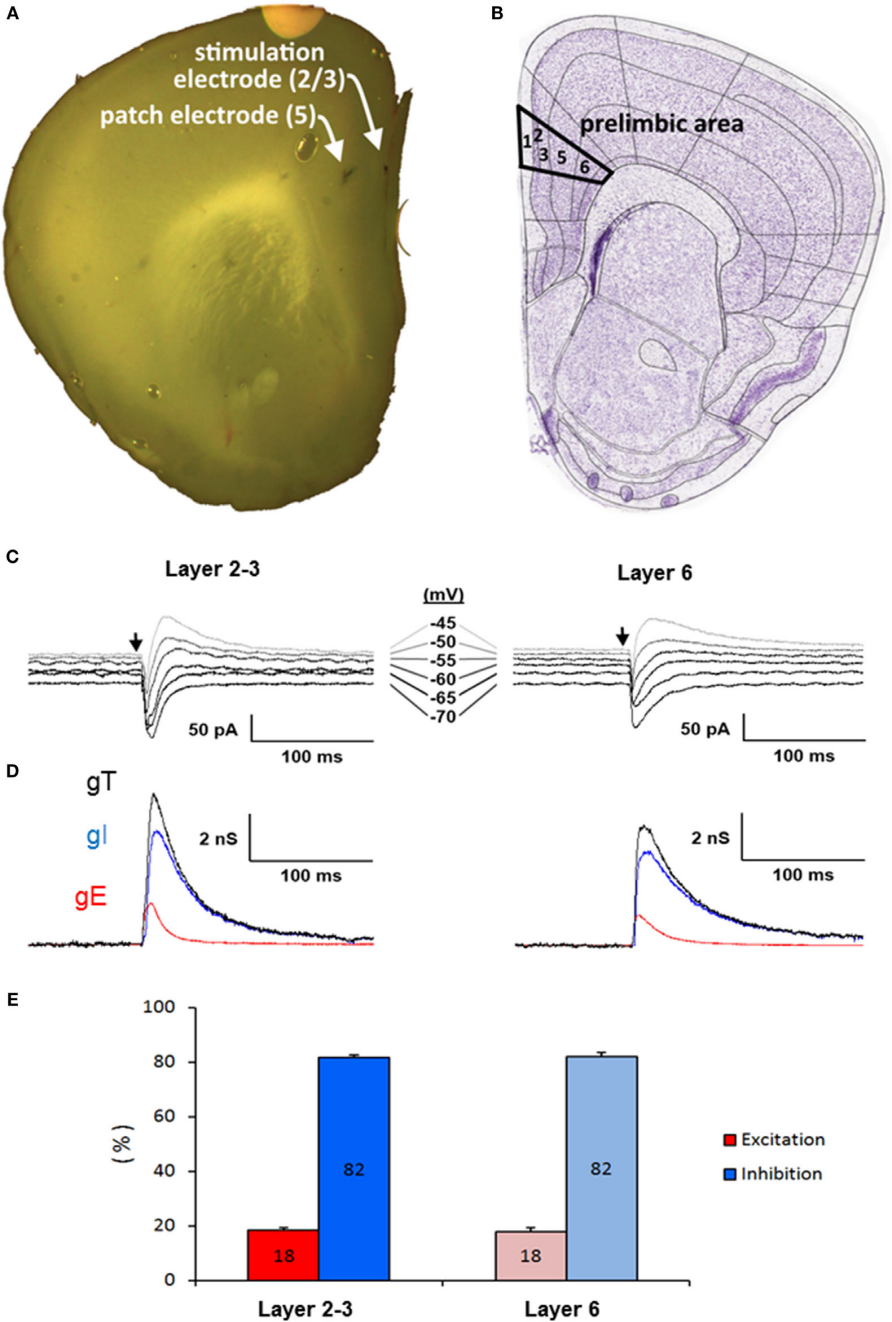
**(A)** Coronal slice of the prefrontal cortex of a 22-day-old male mouse. Arrows show the position of the patch-pipette in layer 5 and of the stimulating electrode in the layer 2-3. **(B)** Scheme of the coronal slice from Allen Brain Atlas Resources Seattle (WA): Allen Institute for Brain Science. ©2009 (Available from: http://www.brain-map.org). **(C)** Representative current responses of a L5PyN to layer 2/3 and 6 stimulation in prefrontal cortex of a WT mouse recorded under voltage-clamp at various holding potentials (each response is the mean of five recordings). Vertical arrows indicate the stimulation onset. **(D)** Corresponding conductance change gT (black line) of the response. Excitatory (gE, dark gray line) and inhibitory (gI, light gray line) conductance changes were obtained from gT decomposition. Data reported are mean ± SD of the mean of *n* layer five pyramidal neurons (L5PyNs). **(E)** E–I balance determined in layer five pyramidal neurons after a stimulation in layer 2–3 (*n* = 25 neurons) or in layer 6 (*n* = 11 neurons).

Stable somatic voltage-clamp recordings of L5PyNs subthreshold postsynaptic responses (composite E–I responses) evoked by layers 2-3 or 6 electrical stimulation (inset A,B Figure 3) were obtained in the PFC and the decomposition method (43) was applied to extract E and I. For each recording (e.g., Figure 3C) the total input conductance (gT) was first extracted (Figure 3D) and its decomposition allowed to further evaluate the relative contribution of evoked excitatory and inhibitory inputs reaching the soma of the recorded L5PyN (Figure 3D). Typical layer 2–3 or 6 electrical stimulation produces a fast excitatory conductance (gE) elicited before a long-lasting inhibitory conductance (gI). Quantification of these somatic conductances showed that the control stimulus-locked composite signal at the soma of L5PyNs is composed of 18% of E and 82% of I whatever the stimulated layer was (Figure 3E, *n* = 25 cells and *n* = 11 cells for stimuli in layer 2–3 or 6, respectively, *p* = 0.8).

**Figure 4:**
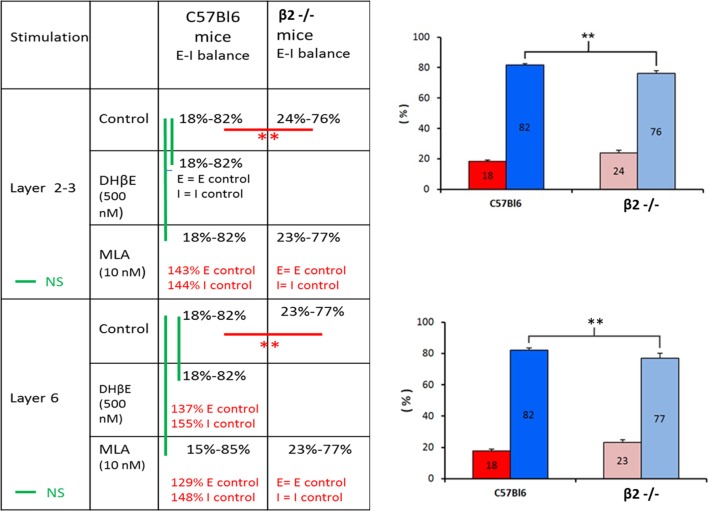
**Effects of DHβE and MLA on the E–I balance**. *Right panel*. Significative changes in the E–I balance determined in layer 5 pyramidal neurons between WT mice and β2^−/−^ mice whatever the location of the stimulus (layer 2-3 or layer 6). *Left panel*. The table summarized the effects of DHβE and MLA on excitatory and inhibitory conductances. Red bars: significative changes in the E–I balance. Green bars: non-significative changes in the E–I balance.

We further explored whether the E–I balance was modulated by ACh around its set-point and to do so we determined the balance in the PFC of β2^−/−^ mice and compared the effects of α4β2 or α7 antagonists on the balance between C57Bl6 mice and β2^−/−^ mice (Figure 4). The E–I balance in β2^−/−^ mice was equal to 24–76% in response to layer 2-3 stimulation (*n* = 16) and to 23–77% in response to layer 6 stimulation (*n* = 6). These values of the E–I balance were significantly different from the values obtained in C57Bl6 mice (*p* < 0.05, Mann–Whitney *U*-test). This result was in favor of a modulation of synaptic inputs on L5PyNs by ACh. Surprisingly, in C57Bl6 mice DhβE (500 nM) the α4β2 nicotinic antagonist had no effect on E and I when the stimulation was applied in layer 2-3 as compared to control condition (*n* = 10, *p* = 0.8). However, the α7 nicotinic antagonist MLA (10 nM) increased E by 43% (*n* = 10, *p* < 0.05) and I by 44% (*n* = 10, *p* = 0.02) without changing the E–I balance (18–82%, *p* = 0.7). In the contrary, MLA had no effect on E and I of β2^−/−^ mice (*n* = 10, *p* = 0.8 for E and *p* = 0.3 for I). We conclude that in superficial layers ACh decreases synaptic inputs on L5PyNs through the activation of α7 receptors and that this modulator effect is lost in β2^−/−^ mice.

The modulation exerted by ACh on synaptic inputs is more complicated in deep layers of the PFC. In C57Bl6 mice, the stimulation of layer 6 in presence of DHβE induced an increase of E by 37% (*n* = 7, *p* = 0.051) and I by 55% (*n* = 7, *p* = 0.057) without changing the E–I balance significantly (*p* = 0.4). Elsewhere, MLA increased E by 29% (*n* = 4, *p* < 0.05) and I by 48% (*n* = 4, *p* < 0.05) with no significant change of the E–I balance (*p* = 0.5). However, in β2^−/−^ mice MLA had no effect on E (*n* = 6, *p* = 0.3) and I (*n* = 6, *p* = 0.5).

Our results showed that the control of excitatory and inhibitory inputs by ACh through α7 receptors was lost in the PFC of mice lacking β2-nAChRs. Moreover, we determined a link between the laminar and cellular segregation of nAChRs and specific functional effects on synaptic inputs on L5PyNs. The change of the modulator effects of α7 receptors in β2^−/−^ mice support the possibility of crossed modifications of expression and function of nAChRs types.

### Experiment II

#### Beta2 Have Alteration in Gambling Task: Mouse Gambling Task

**Figure 5:**
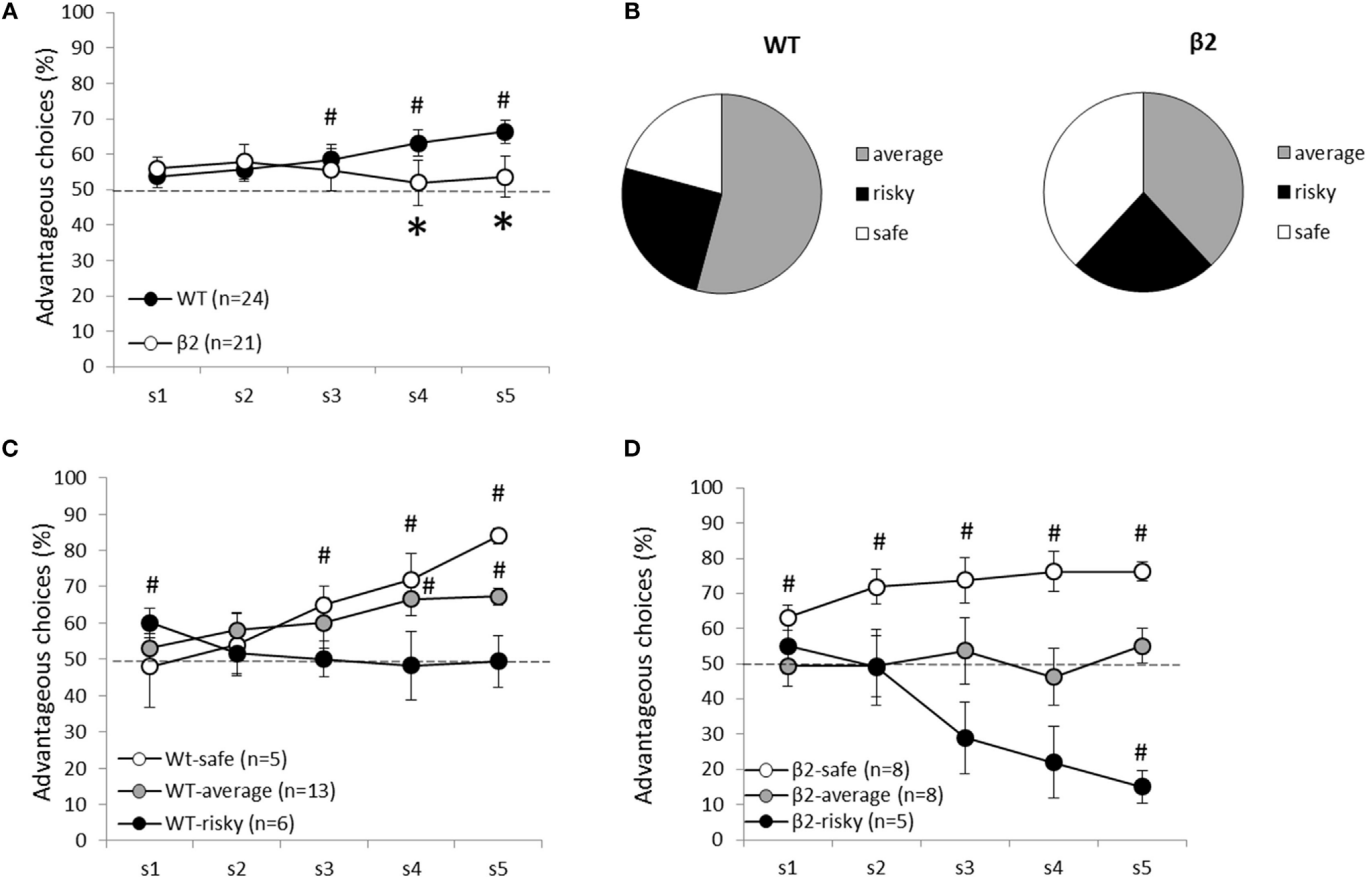
**Animal’s performances during the decision-making task (MGT) are expressed as mean ± SEM for WT and β2^−/−^ mice**. **(A)** Global performances of WT (*n* = 24) and β2^−/−^ mice (*n* = 21) during the MGT. K-means clustering analysis divided each group of mice in three subgroups. Safe, average, and risky mice are represented in **(C)** for WT mice and **(D)** for β2^−/−^ mice. **(B)** Subgroup repartition for each genotype. Significant (*p* < 0.05) difference from chance is represented as # and *represent significant (*p* < 0.05) genotype effect.

As illustrated in Figure 5, mice initially chose equally advantageous and disadvantageous options. Over time, a two-way ANOVA revealed that choice of β2^−/−^ mice and WT mice evolved significantly differently over time as there was a genotype × sessions interaction [*F*_(4, 172)_ = 2.42, *p* < 0.05] with WT favoring advantageous choice [*F*_(4,92)_ = 2.9, *p* < 0.05] while β2^−/−^ mice did not [*F*_(4, 80)_ < 1, ns]. This difference in choice evolution led to a global genotype effect for the last 2 days [*F*_(1,43)_ = 4.43, *p* < 0.05]. Indeed, WT mice chose more advantageous options (Sessions 3, 4, and 5 differed from the chance, Wilcoxon rank sum test, paired: S1 *Z* = −1.120, ns; S2 *Z* = −1.640, ns; S3 *Z* = −2.273, *p* < 0.05; S4 *Z* = −3.071, *p* < 0.01; S5 *Z* = −3.511, *p* < 0.001). By contrast, β2^−/−^ mice were not able to choose advantageous options from disadvantageous ones until the end of the task (S1 *Z* = −1.784, ns; S2 *Z* = −1.784, ns; S3 *Z* = −0.983, ns; S4 *Z* = −0.282, ns; S5 *Z* = −0.678, ns). Choice latencies (data not shown) globally decreased with gambling sessions [*F*_(4,172)_ = 12.28, *p* < 0.05], but this decrease was not the same in the two genotypes (genotype × sessions interaction [*F*_(4, 172)_ = 4.34, *p* < 0.05]). β2^−/−^ choice latencies were shorter than that of WTs at the beginning of the task and were not modified with time [*F*_(4, 80)_ = 2.03, ns]. By contrast, WT mice demonstrated a decrease in choice latency across the five gambling sessions [*F*_(4,92)_ = 14.31, *p* < 0.05]. This differential evolution concerning choice latencies led to a genotype effect restricted on the two first gambling days [*F*_(1, 43)_ = 12, *p* < 0.05].

The k-mean clustering made it possible to separate WT and β2^−/−^ mice in three subgroups of performance: “safe” (WT *n* = 5, β2^−/−^ *n* = 8), “risky” (WT *n* = 6, β2^−/−^ *n* = 5), and “average” (WT *n* = 13, β2^−/−^ *n* = 8). Safe WT animals (Figure 5) developed a preference for advantageous options from the fourth session until the end (S1, S2, S3, ns; S4 *Z* = −2.023, *p* < 0.05; S5 *Z* = −2.023, *p* < 0.05), whereas safe β2^−/−^ mice, already developed a stable preference for advantageous options on the first one session (S1 *Z* = −2.366, *p* < 0.05; S2 *Z* = −2.366, *p* < 0.05; S3 *Z* = −2.310, *p* < 0.05; S4 *Z* = −2.251, *p* < 0.05; S5 *Z* = −2.521, *p* < 0.05). Unlike average WT mice, β2^−/−^ average mice were not able to distinguish advantageous options from disadvantageous ones at the end of the task (average WT S4 *Z* = −2.795, *p* < 0.01; S5 *Z* = −3.059, *p* < 0.01; average β2^−/−^ S4 *Z* = −0.676, ns; S5 *Z* = −1.120, ns). Except for the first session, WT risky mice equally chose advantageous and disadvantageous options throughout sessions (risky WT S1 *Z* = −2.023, *p* < 0.05; S2, S3, S4, S5, ns). Conversely, β2^−/−^ risky mice exhibited a marked preference for disadvantageous options (risky β2^−/−^ S1, S2, S3, S4, ns; S5 *Z* = −2.023, *p* < 0.05). On the last gambling session, there was a significant genotype effect in average (Mann–Whitney: S5 *U* = 0, *p* < 0.05) and risky subgroups (S5 *U* = 30, *p* < 0.01), but not in the safe ones (S5 *U* = 7, ns).

**Figure 6:**
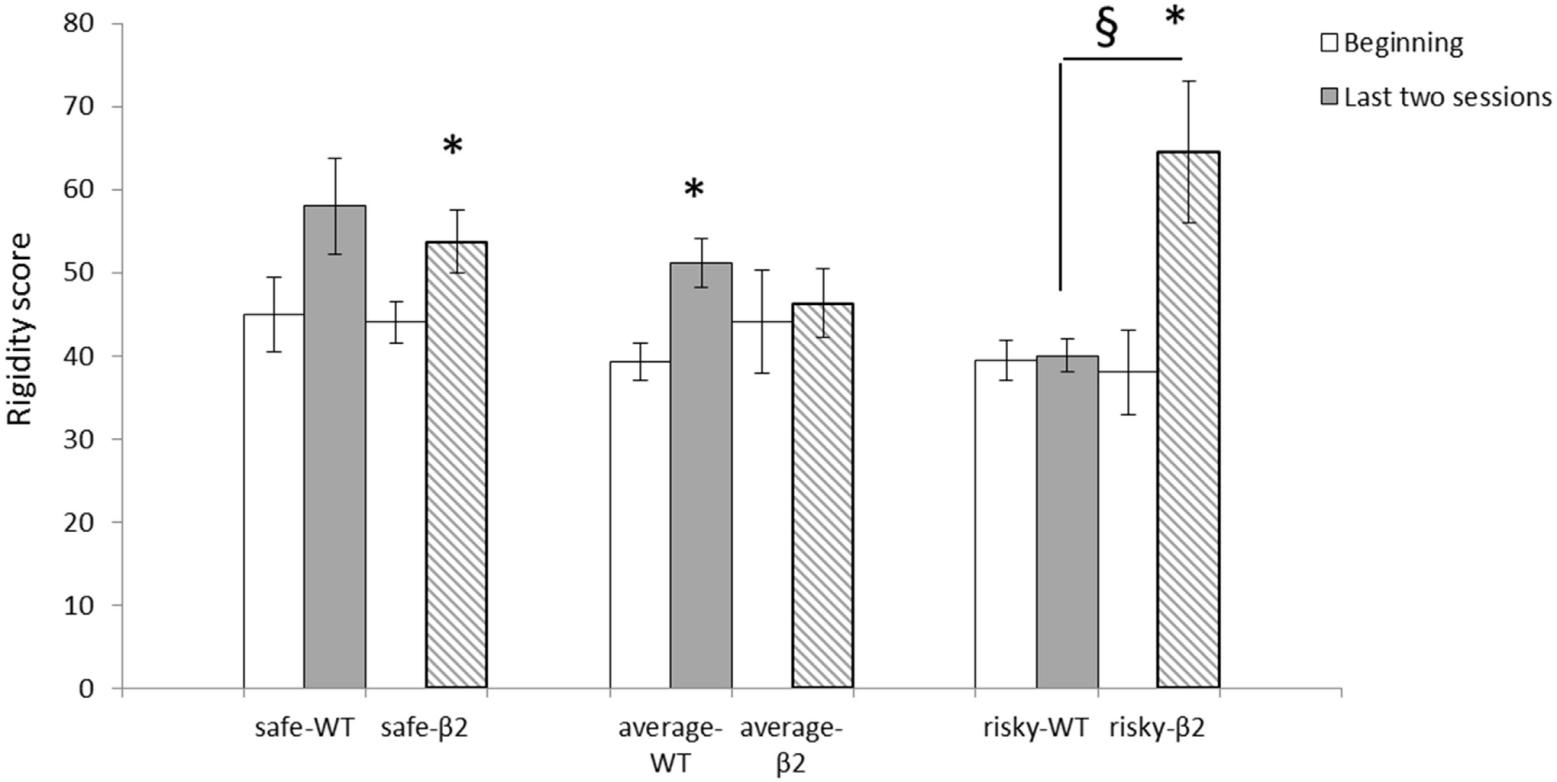
**Rigidity scores at the beginning (sessions 1 and 2) and for the last two sessions (sessions 4 and 5) of the MGT expressed as mean ± SEM for WT and β2^−/−^ mice**. *beginning vs. end of the task *p* < 0.05, § genotype effect *p* < 0.05.

In all animals, rigidity significantly increases from the two first sessions to the last two [*F*_(1)_ = 31.078, *p* < 0.0001]. However, there was no interaction session × genotype [*F*_(1, 1)_ < 1, ns] (Figure 6). There was an effect of session [*F*_(1)_ = 30.44, *p* < 0.0001] and an interaction session × subgroup (safe, average, and risky) for β2^−/−^ mice [*F*_(1,2)_ = 11.28, *p* < 0.001]. For WT mice, however, there was only a session effect [*F*_(1)_ = 22.28, *p* = 0.0001] and no interaction session × subgroup [*F*_(1, 2)_ = 2.55, ns]. The increase of the rigidity score was significantly different for average WT mice (Wilcoxon: *Z* = −3.1, *p* < 0.05) but not for safe (*Z* = −1.461, *p* = 0.1441) or risky mice (*Z* = −0.674, ns). In β2^−/−^ mice, the increase of rigidity was significant for safe (Z = −2.366, *p* < 0.05) and risky mice (*Z* = −2.023, *p* < 0.05) but not for average animals (*Z* = −0.734, ns). Moreover, rigidity scores were significantly different between safe and risky WT mice (Mann–Whitney: *U* = 2, *p* < 0.05), average and risky β2^−/−^ mice (*U* = 6, *p* < 0.05), and between risky β2^−/−^ and WT mice (*U* = 0, *p* < 0.01) during the two last sessions.

#### Differential Activation of Neuronal Circuits in Beta2 vs. WT during Gambling

**Figure 7:**
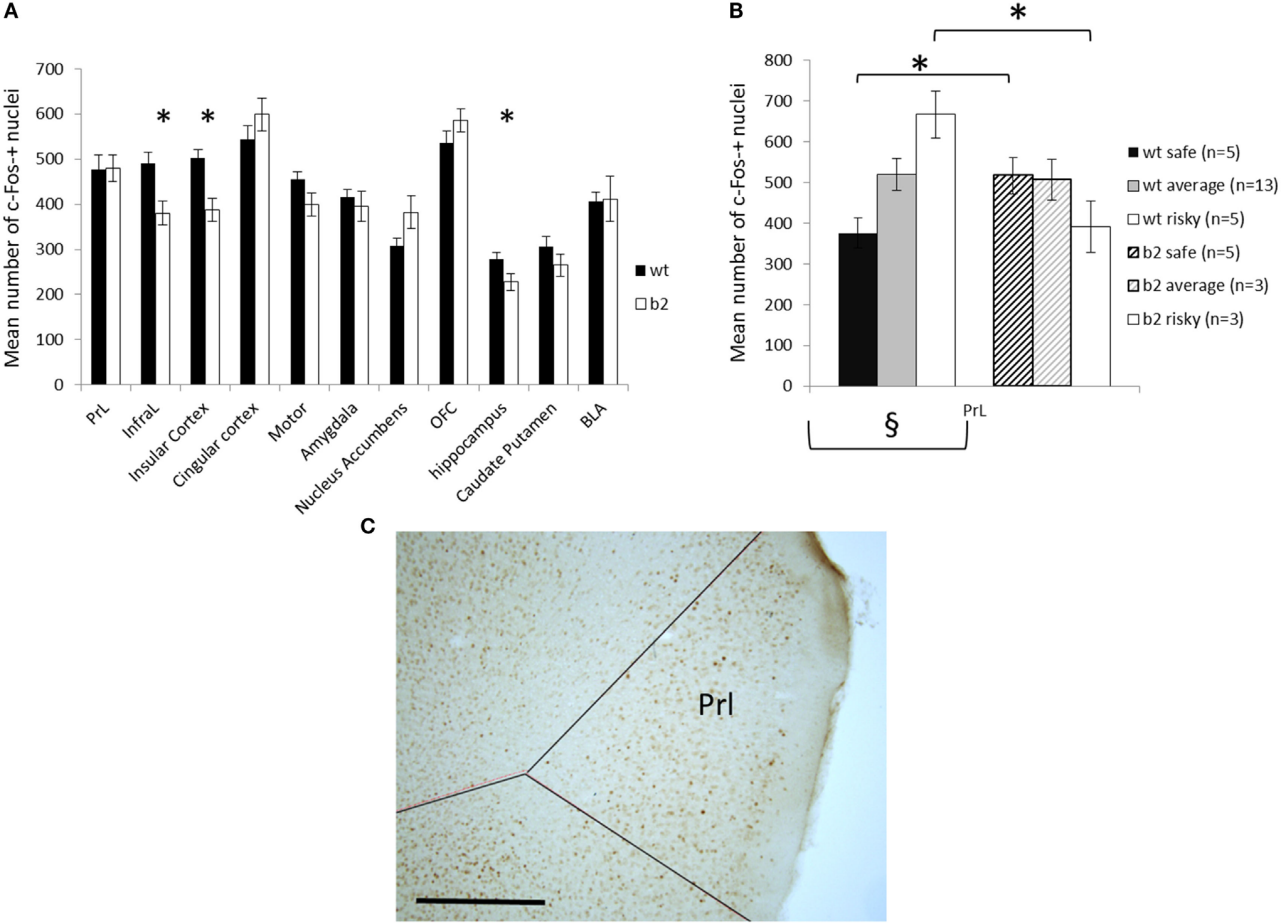
**Cfos expression following the mouse gambling task (MGT) expressed as mean ± SEM for WT and β2^−/−^ mice**. **(A)** cfos expression in the different brain areas. *genotype effect *p* < 0.05. **(B)** cfos expression in the prelimbic cortex in different subgroups of MGT performance (safe, average, and risky) in both WT and β2^−/−^ mice. *genotype effect *p* < 0.05, § significant global subgroup effect in WT mice only. **(C)** Representative microphotography of c-Fos immunohistochemistry in the PrL, scale bar 500 μm.

We measured the brain expression of cFos 90 min after the last gambling session in WT or β2^−/−^ mice allowing us to have an estimation of brain structures activation during the last gambling session (for example of cFos labeling in Prl see Figure 7C). This method demonstrates that β2^−/−^ mice have a significantly lower cFos activation in Infralimbic, Insular cortex, and hippocampus (*U* = 46, *U* = 31, and *U* = 62, respectively, *p* < 0.05). By contrast, all other regions were identically activated in both genotype (Prelimbic cortex, *U* = 103, Cingular cortex, *U* = 96, Motor cortex, *U* = 83, Amygdala, *U* = 93, NAcc, *U* = 79, Orbitofrontal cortex, *U* = 97, CPu, *U* = 87, and BLA, *U* = 101, all ns) (Figure 7A).

For WT animals, cFos expression was significantly different in relation to subgroups only in Prl (Kruskall–Wallis, H = 8.63, *p* < 0.05) and not in all other structures (InfraL, H = 0.58, Cins, H < 1, Cg, H = 1.14, Moteur, H = 0.59, Amy, H < 1, Nacc, H = 2.83, OFC, H = 3.20, Hippocampe, H = 1.06, Cpu, H = 4.56, BLA, H = 2.87, ns). In β2^−/−^ mice, cFos activity was not related to subgroups (Prl, H = 3.39, InfraL, H < 1, Cins, H = 2.45, Cg, H = 2.86, Motor, H < 1, Amy, H < 1, Nacc, H < 1, OFC, H < 1, Hippocampe, H < 1, Cpu, H = 1.74, BLA, H < 1, ns). In Prelimbic cortex, WT “safe” animals demonstrated significantly lower cFos expression than β2^−/−^ “safe” animals (*U* = 0, *p* < 0.05) and WT “risky” animals demonstrated significantly greater cFos expression than β2^−/−^ “risky” (*U* = 0, *p* < 0.05). Average animal display the same cFos expression in Prl whatever the genotype (*U* = 14, ns) (Figure 7B).

### Experiment III

#### Beta2 Have Normal Explicit Choice between Three Natural Motivations

**Figure 8:**
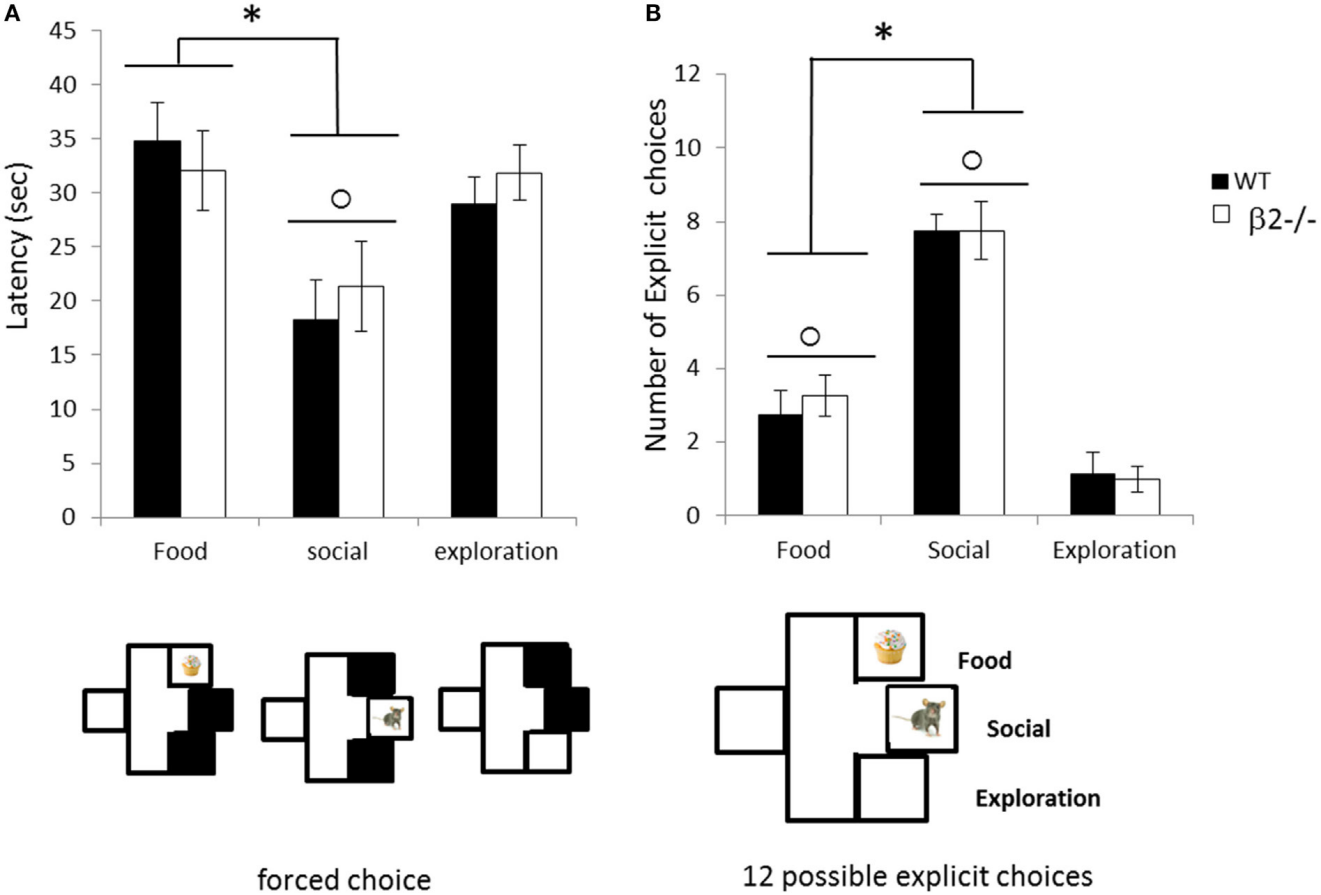
**Choice performance during the explicit choice task expressed as mean ± SEM for WT (black) and β2^−/−^ mice (white)**. **(A)** Mean latency to reach each rewarded box during forced choice. **(B)** Mean number of food (F), social (S) or exploration (E) choice made by animals during the 12 explicit choices. *food vs. social *p* < 0.05, ○ social vs. exploration *p* < 0.05.

Once animals have experienced the reward during the goal exposure, and have been habituated to presence of rewards during 15 min, we assess their motivation for each independent reward during forced choices (Figure 8). During the forced choices, all animals (β2^−/−^ and WT) demonstrated a shorter latency to reach the social goal box in contrast to food or empty one (explo vs. social; Wilcoxon rank sum test, paired, *V* = 131, *p* < 0.001, food vs. social *V* = 132, *p* < 0.001) with no difference between food or exploration goal boxes (*V* = 52, ns). This low latency to reach the social goal was similar in both genotype (genotype effect for Food; Wilcoxon rank sum test, two samples, *W* = 25, ns; Social; *W* = 35, ns; Explo; *W* = 45, ns). During the following explicit choice session, all genotypes clearly choose social goal box in a majority of choices (social vs. food, *V* = 133, *p* < 0.001; social vs. explo, *V* = 0, *p* < 0.001) and they also prefer food goal box over empty box for exploration (*V* = 133, *p* < 0.001) demonstrating a clear ranking of motivation Social > Food > Exploration. Absence of β2 subunit has no significant impact on this ranking (genotype effect for Food; *W* = 36, ns; Social; *W* = 37, ns; Explo; *W* = 35.5, ns).

#### Beta2 Normally Adapt to Change in Motivation

##### Social Devaluation

**Figure 9:**
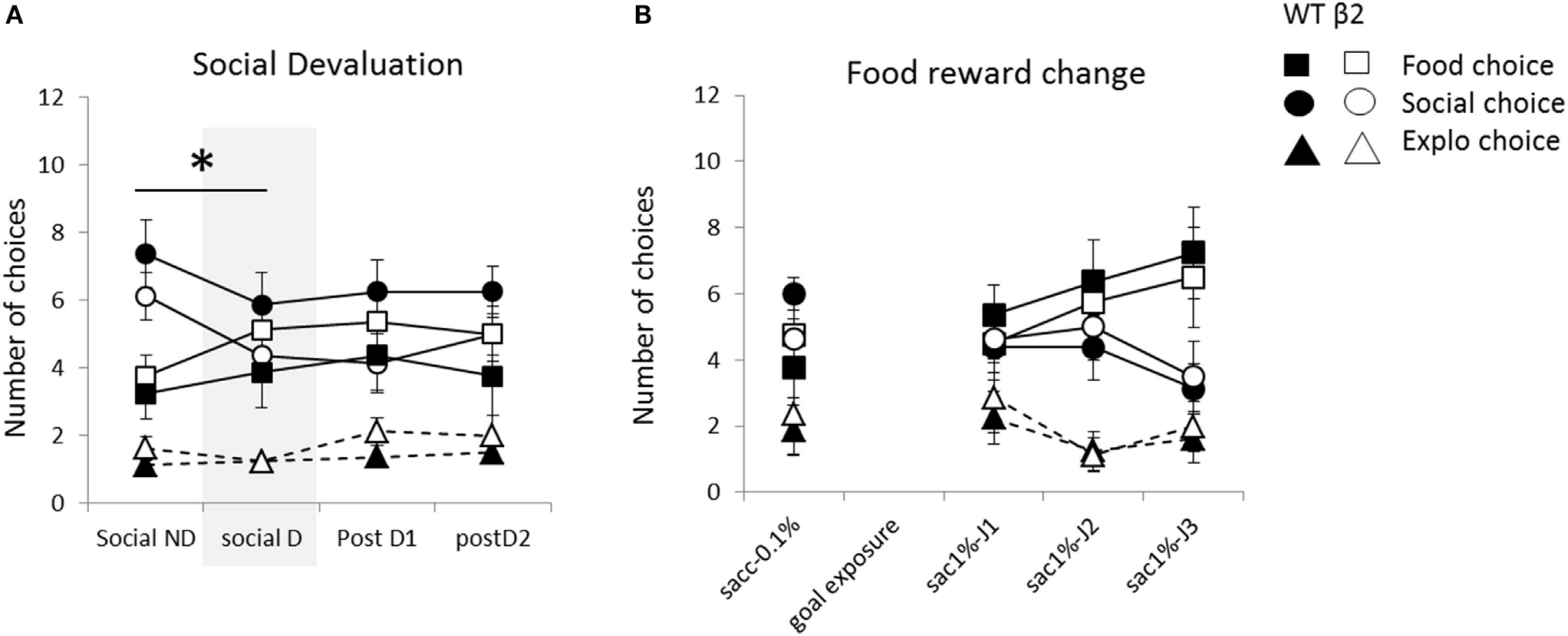
**Animal’s performances during the motivational modulation of explicit choice are expressed as mean ± SEM for WT and β2^−/−^ mice**. **(A)** Mean number of explicit food choice (square), social choice (circle), or exploration choice (triangle) over 12 daily trials. Successive days correspond to successive paradigms applied to all animals: non-devaluation of social reward (Social ND), devaluation of social reward (Social D) and Post D1 and D2 are classic days of 12 explicit choice trials. **(B)** Mean number of explicit food choice (square), social choice (circle), or exploration choice (triangle) over 12 daily trials. Successive days correspond to successive paradigms with increasing number and quality going from one drop of 0.1% saccharine to two drops of 1% saccharin from day sac1%-J1 to sac1%-J3. *p < 0.05. (β2^−/−^ are in white and WT in black).

During the 4 days of social devaluation protocol with social ND, social devaluation (D), and the two following days, only social choice were affected in contrast to others choices (for social choice; Friedman = 8.15, df = 3, *p* < 0.05, for food choice; Friedman = 3.56, df = 3, ns and for explo choice; Friedman = 2.36, df = 3, ns) (Figure 9A). And number of contact to the social mice was also significantly decrease (Friedman = 12.66, df = 3, *p* < 0.01) (data not shown). This significant decrease of social choice and contact is mainly due to a significant decrease between day with devaluation and day without devaluation (for social choice, ND vs. D; *V* = 8, *p* < 0.01, contact; *V* = 30, *p* = 0.05). Moreover, the number of social choice or the number of social contact never came back to non-devalued level with no more evolution on following days (for social choice, evolution between D, and postD1 and postD2; Friedman = 1.08, df = 2, ns; social contact; Friedman = 2.41, df = 2, ns). This decrease in number of social choice and contact, due to devaluation, was unaffected by the absence of béta2 subunit (genotype effect for devalued day; social choice *W* = 20.5, ns; social contact *W* = 21, ns; and for non-devalued day; social choice *W* = 20.5, ns; social contact *W* = 23.5, ns) and there were no genotype effect during following days (social choice: postD1; *W* = 14.5, ns and postD2; *W* = 22, ns; social contact: postD1; *W* = 23, ns and postD2; *W* = 15, ns). Eventually, on the last day (postD2), number of food choice or social choice were equivalent (*V* = 48, ns) and were significantly higher than exploration choice (food vs. explo; *V* = 20, *p* < 0.05, social vs. explo; *V* = 5.5, *p* < 0.01).

Choice latency for food and social constantly decrease during this four days paradigm (data not shown) (for social choice; Friedman = 14.47, df = 3, *p* < 0.01, for food choice; Friedman = 12.375, df = 3, *p* < 0.01 and for explo choice; Friedman = 6.9, df = 3, ns) with no significant difference between D and ND days (for social choice, ND vs. D; *V* = 80, ns; for food choice; *V* = 61, ns) and with no genotype effect on D (social; *W* = 18, ns, food; *W* = 29, ns) and ND days (social; *W* = 34, ns; food; *W* = 26, ns).

##### Change of Saccharin Value and Quantity

We observed a significant rise in number of food choice and decrease in social one from the day with one drop of 0.1% saccharin through 3 days with two drops of 1% saccharin (food choice; Friedman = 9.13, df = 3, *p* < 0.05; social choice: Friedman = 9.08, df = 3, *p* < 0.05) with no evolution of choice of empty box (Friedman = 5.26, df = 3, ns) (Figure 9B). During these days, increasing the value and quantity of food reward significantly decreases latency to reach the food goal box but also the social one (Friedman = 11.1, df = 3, *p* < 0.05; Friedman = 20.92, df = 3, *p* < 0.001, respectively) with no genotype effect (social latencies *W* = 23, 39, 32, and 13, ns; food latencies, *W* = 33, 29.5, 41, and 43, ns). Latency to enter the empty box would not be analyzed on following manipulations due to the insufficient number of empty choice, which prevent us to have relevant latency. Animal go from a ranking of choices with social choice higher than exploration (*V* = 17.5, *p* < 0.01) and equivalent to food (*V* = 27.5, ns) to ranking with a predominant choice for food over social or exploration (respectively *V* = 98, *p* < 0.05 and *V* = 18.5, *p* < 0.05). Even with this predominant increase of food choice, social choice number is still significantly higher than exploration one (*V* = 18.5, *p* < 0.05). On the first day before the shift, β2^−/−^ mice demonstrated same choice for social box (*W* = 14, ns) with significantly less number of social contact (*W* = 12, *p* < 0.05) and no impact on food or exploratory choice (*W* = 40.5, ns; *W* = 32, ns). However, β2^−/−^ adapt their choice in similar manner than WT mice (genotype effect on food choice for sac J1-2-3, respectively, *W* = 23.5, 26, and 28.5; ns; on social choice for sac J1-2-3, respectively, *W* = 33, 40.5, and 34.5). interestingly, during these 3 days, the mean number of social contact on these 3 days with novel reward is significantly lower in β2^−/−^ than in WT (stat on the mean of the three days: *W* = 9.5, *p* < 0.05; WT, mean of 6.06 ± 0.44 contact, β2^−/−^ mean of 4.78 ± 0.23).

#### Food Devaluation

Devaluation of food has no significant effect on food, social, or exploratory choice (*V* = 50, ns; *V* = 49, ns and *V* = 25.5, ns) nor on social contact (*V* = 65, ns). Moreover, genotype demonstrate the same kind of choices in non-devalued (food, *W* = 28.5, ns, social, *W* = 36, ns; explo, *W* = 33.5, ns) or devalued day (food, *W* = 28, ns, social, *W* = 34.5, ns; explo, *W* = 33.5, ns). However, food devaluation significantly increases latency to reach the food box (*V* = 118, *p* < 0.01) but not latency for social choice (*V* = 85, ns). This impact of devaluation on latency was similar for WT or β2^−/−^ mice (WT vs. β2^−/−^, food latency on D; *W* = 26, ns on ND, *W* = 25, ns; social latency on D, *W* = 33, ns, on ND; *W* = 27, ns). As in the previous manipulation, β2^−/−^ mice have a trend to demonstrate less social contact than WT (on D day, *W* = 12, *p* < 0.05, ND day, *W* = 15.5, ns, on the mean of both day *W* = 7, *p* < 0.01).

##### Beta2 Have Alteration in Adaptation to Rule Change in Extinction

**Figure 10:**
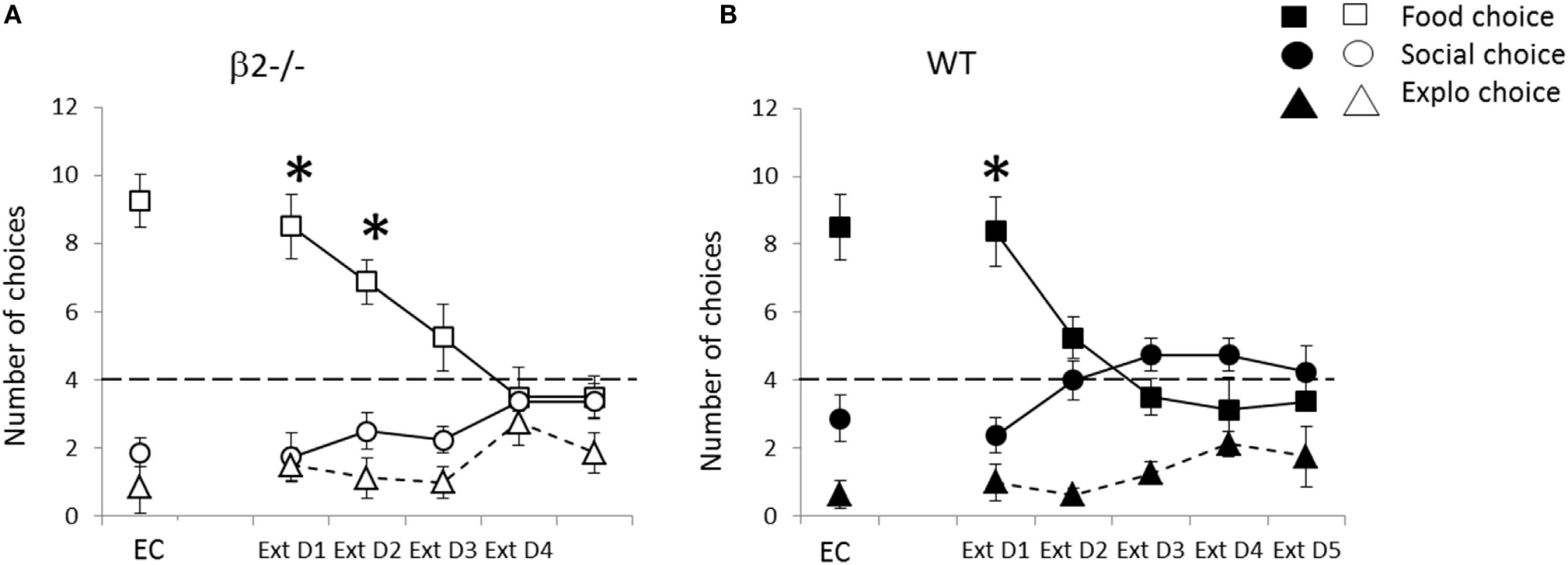
**Performance during the rule change session during extinction expressed as mean ± SEM for WT (B) and β2^−/−^ mice (A)**. Successive days correspond to baseline explicit choice day (EC) and following days are extinction days applied to all animals: mean number of explicit food choice (square), social choice (circle), or exploration choice (triangle) over 12 daily trials *p < 0.05 food vs. social choice. Dashed line represents level of equivalent choices (chance level) between the three available options.

When all rewards were removed, animals significantly decrease their choice to the previously food rewarded box, i.e., ex-food (Friedman = 32.93, df = 4, *p* < 0.001) and increase their choice to the previously social rewarded box, i.e., ex-social (Friedman = 19.90, df = 4, *p* < 0.001) (Figure 10). They also slightly increase their choice toward previously empty box (Friedman = 12.55, df = 4, *p* < 0.05). When look carefully, these evolutions drive the choice of all animals from food predominance (Extinction D1; food vs. empty, *V* = 0, *p* < 0.001, social vs. food *V* = 4.5, *p* < 0.01, and empty vs. social *V* = 24.5, ns) toward almost equivalence of all empty boxes, i.e., four choice in each one, but with still a tendency to ExtD5; food vs. empty, *V* = 31, *p* = 0.057 and empty vs. social *V* = 13.5, *p* < 0.05 and no more difference between ex-food and ex-social (*V* = 71.5, ns). On the graph, we see that evolution of choices is slower in β2^−/−^ mice leading to a conserved difference between choice in ex-food and ex-social on second day compare to WT (ExtD1: ex-food vs. ex-social; β2^−/−^, *V* = 0, *p* < 0.05, WT; *V* = 1.5, *p* < 0.05; ExtD2, ex-food vs. ex-social; β2^−/−^, *V* = 0, *p* < 0.05, WT; *V* = 4.5, ns) and a trend on third day (ex-food vs. ex-social; β2^−/−^, *V* = 4, *p*-value = 0.057, WT; *V* = 18, ns). This slowing down due to genotype appears significant only for ex-social choice on extinction days 3 and 4 (*W* = 3.5, *p* < 0.01, *W* = 13, *p* < 0.05). Moreover, during these 5 days of extinction, latency to choose ex-social and ex-food significantly increased for both genotype (ex-food, Friedman = 44.85, df = 4, *p* < 0.001; ex-social, Friedman = 19.01, df = 4, *p* < 0.001).

## Discussion

In this paper, we clearly demonstrate that β2 nicotinic acetylcholine receptors (β2-nAChRs) within the prelimbic area of the prefrontal cortex are major actors influencing E–I balance. Using β2^−/−^ mice, we demonstrate that the value of the E-I balance was significantly elevated compared to WT mice (E–I, 18–82% in WT to E–I, 24–23% to 76–77% in β2^−/−^). Our results also show that the control of excitatory and inhibitory inputs by ACh through α7 receptors is lost in the prelimbic cortex of mice lacking the nicotinic β2 subunit.

Previous measurements of E–I balance had been successfully used to show the effect of ACh or serotonin in the rat visual cortex (44, 45) and in the mouse PFC (37, 38). Here, we show that the E–I balance (18–82%) in the C57Bl/6 strain was not significantly different from the E–I balance (20–80%) in the PFC of 129/Sv mice (38). This result shows that coordinated functions of neuronal networks regulate the E–I balance of synaptic inputs on layer 5 pyramidal neurons (L5PyNs) in the PFC of C57Bl/6 mice similarly to other mouse strains, and this is crucial for keeping neuronal networks of the PFC in a functional range.

Our results also show that the control of excitatory and inhibitory inputs by ACh through α7 receptors is lost in the prelimbic of β2^−/−^ mice. α7-nAChRs are highly involved in the development of cortex and disruption of their function might lead to neurodevelopmental disorders, such as schizophrenia or other psychiatric disorders (46). Moreover, α7-nAChRs play a major role in the development of cortical parvalbumin-containing GABAergic interneurons (47). Thus, absence of α7 regulation in the PFC of β2^−/−^ mice might lead to alteration in the wiring of inhibitory circuits within the PFC and altered PFC functioning. Additional studies are necessary to decipher the exact roles of β2 vs. α7 in the regulation and development of PFC E/I balance.

Alteration (increased excitation and decreased inhibition) of E/I balance was measured in adolescent β2^−/−^ mice, while decision-making defects were evidenced in adults. We can, thus, wonder whether the E/I prefrontal alteration during development led to an altered prefrontal functioning and wiring which itself had consequences at adulthood, or whether the altered E/I balance plays a direct role in adulthood and impairs prefrontal functioning *per se*. One argument toward an effect not only during development is the fact that viral re-expression of β2 subunit in the PFC of β2^−/−^ mice was sufficient to restore social interactions (28). Interestingly, optogeneticaly mediated elevation of the PFC E/I balance in adult mice was shown to decrease social choice (20) and conditional neuroligin-2 knockout adult mice exhibited a reduction of PFC inhibition associated with altered social interactions (48). We, thus, might suggest that PFC E/I balance modifications in β2^−/−^ mice remain such at adulthood and may be at least partially responsible for decision-making alterations both social and non-social situations. This remains at this point only speculative. It would, however, be of interest to measure individual E/I balance in animals previously subjected either to the gambling task or to the social choice task.

We demonstrate here an involvement of β2-nAChRs in MGT in which uncertainty and risk have to be managed as outcomes are probabilistic. Indeed, β2^−/−^ mice were not able to choose long-term advantageous options from disadvantageous ones until the end of the task. This choice profile led β2^−/−^ mice to make largely less advantageous choices than WTs. As previously reported (8), a majority of WT mice (54%) preferred advantageous options without neglecting alternative but rare – potentially more risky – choices, i.e., *average* mice. A small subgroup of mice (21%) continued throughout the experiment to explore all available options despite a putative risk, i.e., *risky* mice. Another small proportion of mice (25%) strongly preferred long-term advantageous choices, avoided exploring alternative options and presented a more rigid behavior compared to the others, i.e., *safe* mice. β2^−/−^ mice could also be classified in three subgroups but evolution of their choices across sessions was very different from that showed by WTs. Indeed, the β2^−/−^ average mice did not prefer the advantageous options at the end of the task; they had the same percentage of advantageous choices than WT risky mice at the end of the task. Moreover, risky β2^−/−^ mice showed a marked preference for disadvantageous options. To that regard, they had the same profile of choice than poor performance of human patients with bilateral lesions of the ventromedian prefrontal cortex (vmPFC) (39, 49).

Furthermore, mice distribution between the three subgroups was quite distinct from that of WTs: there was a similar proportion of safe and of average mice (i.e., 38%) while 24% of the mice belonged to the risky subgroup. As a result, the absence of β2-nAChRs led mainly to extreme profiles, with no real average subgroup and only safe and risky mice. In addition, a new behavioral profile appeared as some mice strongly preferred disadvantageous options. It is noticeable that the rigidity score of WT mice was roughly similar to that observed previously (8), and particularly that it increased across sessions. This increase reflects the establishment of a fixed choice pattern, away from exploration of multiple options. Average β2^−/−^ mice, however, did not show any increase in rigidity scores across sessions, thus supporting the idea that β2^−/−^ mice behaved like the risky WT mice and continued to explore available options until the end of the task. Risky β2^−/−^ mice increased strongly their rigidity score at the end of the task by choosing nearly exclusively disadvantageous options. We never observed such extreme profile in WT mice (4, 8). Multiple factors might explain choice profiles of β2^−/−^ mice, like alteration in sensitivity to punishment/risk-taking and/or flexibility.

It was proposed that vmPFC patients could either be more sensitive to reward, or insensitive to punishment, or insensitive to future positive, or negative consequences (49). Moreover, vmPFC patients increased betting regardless of the odds of winning during the Cambridge Gamble Task (CGT) a task for which probabilities to loose are presented explicitly (50). Interestingly, patients with insular cortex lesion also failed to adjust their bets by the odds of winning (50). The latter study indicated a necessary role of the vmPFC in decision-making regulation and of the insular cortex in the signaling of aversive outcomes (50).

Here, we observed that β2^−/−^ mice had a hypoactivation of the infralimbic (IL) and insular (CIns) cortices, and of the hippocampus (H). The IL cortex was proposed to be the functionally equivalent to the vmPFC in humans (51). Altogether, these data supported that in β2^−/−^ mice hypoactivation led to poor MGT performance because of a difficulty to regulate decision-making (IL) and to integrate the value of negative outcome (CIns). During the forced and explicit choice task no negative outcome existed. Likewise, during the food or social devaluation task there was no negative outcome. Conversely, during the extinction task mice were not presented with the reward, which could be perceived as a negative condition. Therefore, the slower evolution of β2^−/−^ mice choices during the extinction task could be linked to the hypoactivation of CIns, hence, to a difficulty to detect changes in outcomes. At the level of prelimbic cortex, in which β2^−/−^ mice displayed E/I balance alteration, cfos activation of β2^−/−^ mice was not related to gambling performance. This contrasted with WTs’ c-fos activity for which higher expression correlated to lower rigidity scores. Thus, poor performance of β2^−/−^ mice might be linked to differential activation of neuronal circuits including, IL, PL, CIns, and hippocampus.

It was previously demonstrated that β2^−/−^ mice were hyperactive while displaying less exploratory behavior compared to WT animals (27, 30–32). Our current results showing reduced choice latency in gambling remind our previous data (26) and might be related to the unbalanced locomotion/exploration previously shown to be controlled by nAChRs activity on dopaminergic neurons of the substantia nigra pars compacta (SNpc) and ventral tegmental area (VTA) (27). It was suggested that decision-making processes result in a balance between exploiting existing options and exploring new possibilities (52), with a main involvement of dopamine (DA) in cortico-striatal circuits. Thus, it may be that β2^−/−^ mice that are less explorative are more prone to favor the exploitation of a chosen strategy during the MGT, thus resulting in more extreme profiles, and increasing rigidity. In β2^−/−^ mice, exploration was restored with re-expression of subunit in VTA and not SNpc, suggesting role of nAChRs in accumbal and prefrontal DA input (27). In β2^−/−^ mice, alteration in basal levels of dopamine and serotonin in fronto-striatal circuits (25, 31) might have altered the valuation process when different rewards compete. Indeed, dopamine signaling in the prelimbic cortex plays a major role in goal-directed behavior and ability to detect motivational value of outcomes (53), as well as in selective attention of cues predicting reward (54). Previous data (29) and current results clearly demonstrate that β2^−/−^ mice may adapt normally their behavior when the choice to be made is essentially underpinned by motivational value of outcome with no uncertainty or risk involved. This strongly suggests that decision alteration seen in gambling task in β2^−/−^ mice was not due to a valuation or motivation processes deficit *per se*. We, thus, suggest that dopamine alteration in fronto-striatal circuits of β2^−/−^ mice may underpin, at least in part, decision-making alteration seen in the MGT. Accordingly, the fact that β2^−/−^ mice showed perseveration in extinction task together with the well demonstrated role of prelimbic cortex in flexibility (28, 34) suggests that gambling alterations of β2^−/−^ mice are due to prefrontal dysfunction leading to lower exploration and higher rigidity.

## Conclusion

In conclusion, we demonstrate for the first time that β2-nAChRs play a critical role in the fine tuning of prefrontal E/I balance and that lack of these receptors change α7-mediated prefrontal activity modulation. A shifted set-point of the E/I balance may promote dysfunction of infralimbic, prelimbic and insular cortices and of hippocampus, behaviorally leading to decision-making defects, at the origin of which are lack of flexibility and blunted sensitivity to punishment, specifically when uncertainty regarding outcome is high.

## Author Contributions

SG designed the gambling task, supervised the behavioral experiments and their analyses, and wrote the paper. AF conducted the social task experiments, performed statistical analyses, and wrote the paper. EP, AC, and EM conducted the gambling task, performed statistical analyses, and wrote the paper. XL and CM performed the electrophysiology experiments and analyzed the data. PF designed the electrophysiology experiments, analyzed the data, and wrote the paper. AR supervised the behavioral experiments and wrote the paper.

## Conflict of Interest Statement

Dr. AC is the head of a CRO dedicated to behavioral research. The remaining authors declare that the research was conducted in the absence of any commercial or financial relationships that could be construed as a potential conflict of interest.
